# Zinc oxide nanoparticles promote the aging process in a size-dependent manner

**DOI:** 10.1007/s10856-021-06602-x

**Published:** 2021-09-30

**Authors:** Mahla Deylam, Effat Alizadeh, Manizheh Sarikhani, Marzie Hejazy, Masoumeh Firouzamandi

**Affiliations:** 1 Department of Pathobiology, Faculty of Veterinary Medicine, University of Tabriz, Tabriz, Iran; 2 Department of Medical Biotechnology, Faculty of Advanced Medical Sciences, Tabriz University of Medical Sciences, Tabriz, Iran; 3 Department of Basic Science, Faculty of Veterinary Medicine, University of Tabriz, Tabriz, Iran

## Abstract

Zinc oxide (ZnO) nanoparticles (NPs) are generally utilized in cosmetic goods, sheds, biosensors, and delivery of drug. As in vitro ideal systems, mesenchymal stem cells (MSCs) are used to test acute toxicity. In the present study, size-dependent cytotoxicity effects of ZnO NPs on MSCs were assessed. Bone marrow and adipose MSCs were treated with ZnO NPs with average sizes of 10–30 and 35–45 nm. The 5 and 10 µg/ml concentrations of ZnO NP were found to be the safe concentrations for the NP sizes of 10–30 and 35–45 nm, respectively. Cell-cycle analysis indicated that the small size of ZnO NPs has more negative effects on the process of cell entry to DNA synthesis when compared to the larger size. The results of the β-galactosidase test showed the promotion of the aging process in the cells treated with the smaller size of ZnO NPs. Both sizes of the NP were found to upregulate the aging-related genes NF-kB and p53 and downregulate the anti-aging gene Nanog. To sum up, the smaller size of ZnO NPs can enhance the aging process in the cells.

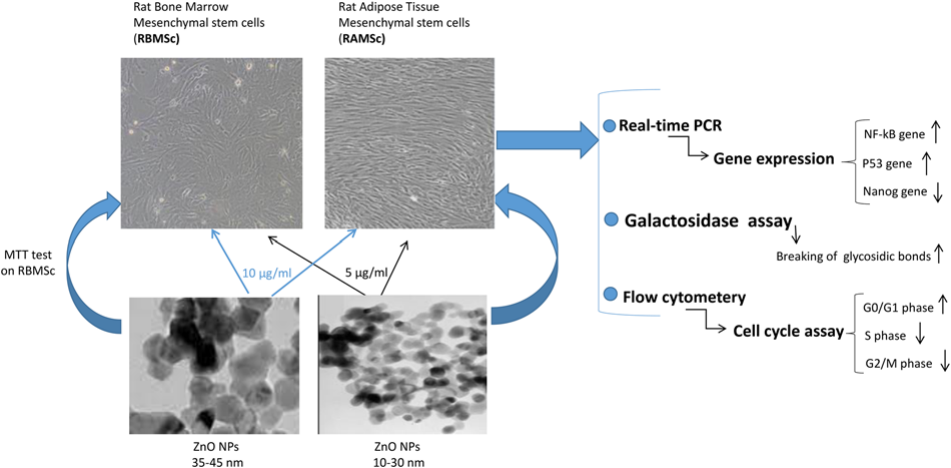

## Introduction

In the recent decade, the nanotechnology industry needs to be developed quickly around the world. Zinc oxide (ZnO) nanoparticles (NPs) are generally utilized in beauty care products, sheds, biosensors, drug delivery, bioimaging, and as antifungal and antibacterial agents [1–5]. ZnO nanostructures have a few excellent properties as well as compound stability, great specific surface range, high electron correspondence highlights, and electro compound activity [6]. ZnO NPs are for the most part used as a UV light dissipating added substance in cosmetic products such as sunscreens, toothpastes, and magnificence products [7, 8]. With the widespread application of nano-structured substances for commercial items, the biosafety of these materials has been considered to explore the natural and toxicological effects of aberrant and immediate manifestation of these substances [9]. Owing to their reactive oxygen species (ROS) and insoluble metal ions, nanomaterials such as ZnO have toxic impacts on the cells [10, 11]. As the toxicity of ZnO NPs is higher in ultrapure water than in phosphate-buffered saline (PBS) in various aqueous media, the toxicity of ZnO NPs is mostly in line for the free zinc ions [12]. While zinc NP complex with media and cause lower toxicity, the concentration of Zn^2+^ ions is greatly reduced. The generated Zn^2+^ insoluble ZnO NPs induce necrosis and inflammation [13]. Intercellular ROS, which is caused by ZnO NPs, induces cell death and dysfunction of mitochondrial oxidative phosphorylation [10].

Several in vivo experiments have indicated the harmful effects of Zn NPs on various life forms such as drosophila [14], fish [15], amphipods [16], mice [17], rats [18], and bacteria [19]. Zn NPs have also been reported to show in vitro toxicity [20–22]. Despite many hidden toxicities associated with ZnO NPs, they are widely used for various biomedical applications and pharmaceutical purposes [23]. Hence, further research is needed to evaluate the toxic impact of this compound on the cells. In toxicology research, MSCs are utilized as an ideal in vivo modeling [24]. Isolation and extension of MSCs in culture is easy, and their differentiation is done through proper stimulation. Bone marrow mesenchymal stem cells (BMSCs) show sensitivity to cytotoxicity tests of cancer medicines and some other cytotoxic medications [25]. Moreover, adipose-derived mesenchymal stem cells (AMSCs) are utilized to assess drug safety and to discover medications [26]. Therefore, in this study, we supposed that AMSCs and BMSCs targeted by ZnO NPs might be suitable for considering the potential risks in the aging development. To evaluate cell toxicity, gene expression assay, a factor that plays a role in early toxic processes, is highly significant [26]. Thus, as a specific stem cell marker, Nanog gene was utilized in the present research to anticipate toxicity. Nanog has two functions in stem cell differentiation and self-renewal [27]. Moreover, Nanog gene stimulates the senescence suppression by downregulating the expression of p27^KIP1^ gene [28]. In classic response to genotoxicity, to restrict cell proliferation, p53 in the corrupted genomes causes the cell-cycle arrest and cell death. Numerous investigations have highlighted the role of NF-kB framework in activating the stimulating changes in tissues during aging [29]. Therefore, in the present study, cell-cycle assay, galactosidase in situ assay, and mRNA levels of NF-kB, p53, and Nanog genes were considered.

## Materials and methods

### Properties and characterization of nanoparticles

**Fig. 1:**
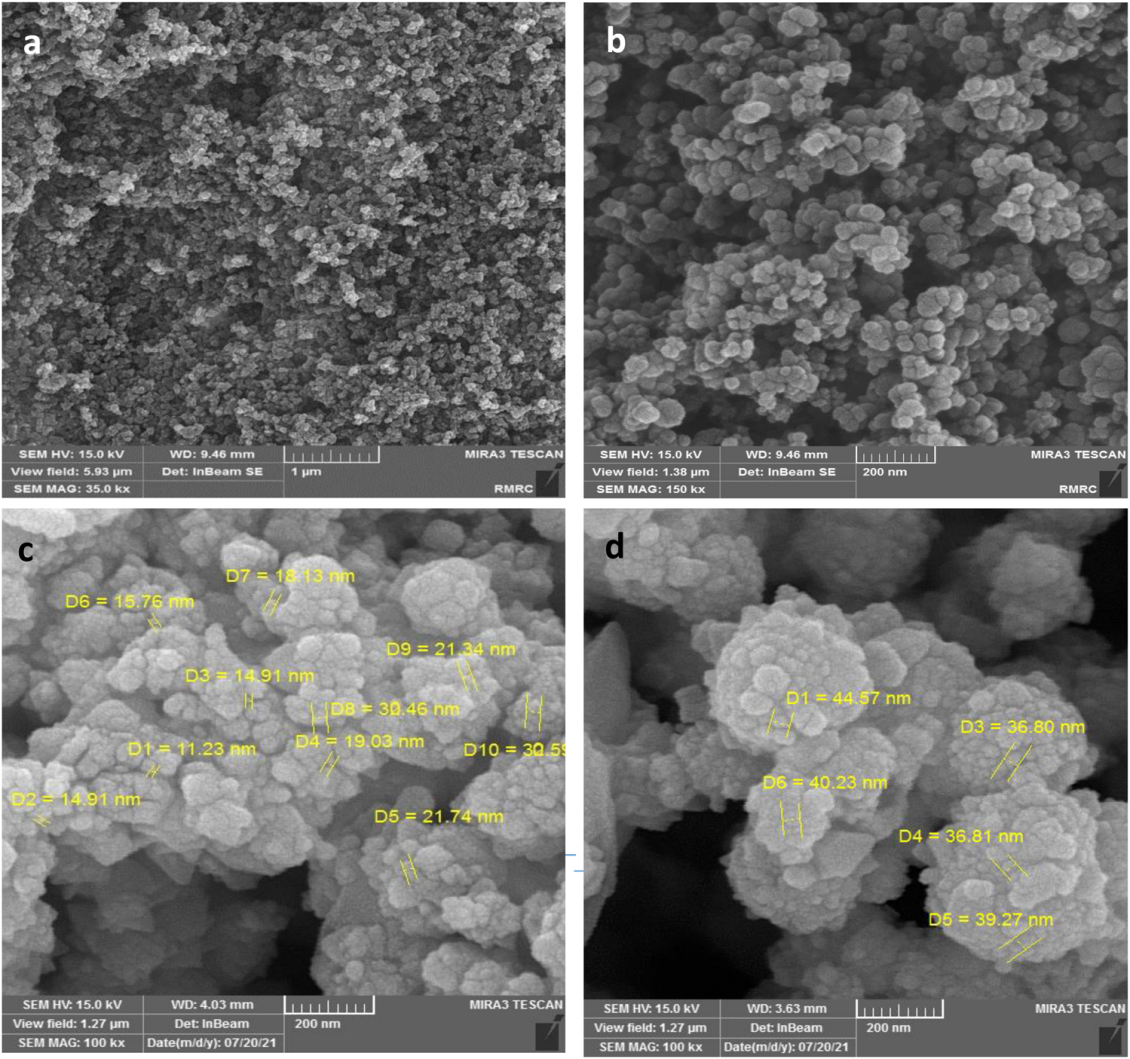
SEM images of ZnO nanoparticles. **a** ZnO NPs in 1 µm scale, **b** ZnO NPs in 200 nm scale, **c** size of 10–30 ZnO NPs in 200 nm scale, **d** size of 35–45 ZnO NPs in 200 nm scale

Two different sizes of ZnO NPs (‏Sigma Aldrich, USA) with the following information were purchased: one with 10–30 nm average size, +99% purity, 5.606 g/cm^3^ density, and about 20–60 m^2^/g surface area, and the other with 35–45 nm average size, +99% purity, 5.606 g/cm^3^ density, and about 65 m^2^/g surface area (Fig. 1). Then, a stock of 100 µg/ml of ZnO NPs suspension in 1 ml of PBS was prepared by sonication for 3 min.

### Isolation of mesenchymal stem cells

BMSCs were picked from 8-week-old (200–250 g) male rats. The epiphyses were removed, the marrow cavities were accessed, and total bone marrow (BM) plugs were blushed out of tibial and femoral bones by a 10 ml syringe containing Dulbecco’s modified eagle medium (DMEM) (Laboratories Inc., MA, USA) plus 10% fetal bovine serum (FBS). BM samples were gathered and precisely upset by successive desire needles of 18 and 20 gauge appended to a similar 10 ml syringe. Then, the cell suspension was centrifuged for 5 min at 1000 rpm. After pelleting the cells, they were resuspended in the medium with 10% FBS. To play out a cell tally counter, 4% acetic acid was mixed with a low amount of the suspension to lyse the red blood cells. Counting of the cells was done by hemocytometer. Subsequently, the 5 × 10^7^cells cells were plated in 100 mm culture dish, kept in 5% CO_2_ at 37 °C, and changed with a fresh medium each 3–4 days [30]. Using collagenase type I (0.15% weight to volume) for 1 h at 37 °C, the adipose tissue was then enzymatically separated. To remove undissociated particles, the suspension was filtered with a 70-μm filter. Then, DMEM with 10% (v/v) FBS was added and centrifuged at 700 × g for 5 min. Finally, the pellet of the cell was resuspended in DMEM with 1% (v/v) penicillin/streptomycin and 10% (v/v) FBS [31].

### Cell culture

AMSCs and BMSCs were cultured in DMEM (Laboratories Inc., MA, USA) with 1% antibiotics and 10% FBS in the standard culture conditions (at 37 °C, in 5% CO_2_, and 95% humidity) [32].

### Characterization of surface markers of BMSCs and AMSCs

BMSCs and AMSCs were harvested for 5 min at 37 °C, plated by 200 × g centrifugation for 5 min, and rinsed with chilled PBS. Next, 5 µl of fluorescein isothiocyanat (FITC)-conjugated antibodies, including CD34-PE, CD90-FITC, CD45-FITC, and CD73-FITC (Thermo Fisher Scientific, Germany), was added to the BMSCs and AMSCs and then stored at room temperature (RT) and a dark place for 20 min. The samples were evaluated with a flow cytometer (BD FACS Caliber; San Jose, CA, USA) [33, 34].

### In vitro cytotoxicity

For cytotoxicity testing of NPs, the BMSCs and AMSCs were culture into 96-well plates at confluency of 70–80%, all particles were suspended in complete DMEM (10% diluted NPs with 90% DMEM containing PBS) [35]. Bath sonication was done twice, each time for 5 min, to finely mix the final ZnO NPs concentrations. Therefore, the BMSCs and AMSCs were treated with different concentrations (0, 3, 5, 10, 25, and 50 µg/ml) of ZnO NPs for 24, 48, and 72 h. Then, MTT assay was used to test the viability of the treated cells. Preparation of MTT stock solution ((3-(4,5-diminish ethylthiazol-2-yl)- 2,5-diphenyltetrazolium bromide) was done by adding 1 ml PBS to 5 mg MTT (Sigma, USA) in a dark place. Then, 20 µl of the stock solution was mixed with all experimental wells (control cells and ZnO NPs treated with different concentrations), vibrated for 10 min, and incubated at 37 °C for 3 h. Lastly, the samples were ousted from the incubator and mixed with DMSO, with a purple color noticeable at this stage. They were developed over pipetting and read immediately in the presence of UV at 570 nm.

### Cell-cycle assay

BMSCs and AMSCs were seeded in different 6-well plates. While the 70% cell confluence was observed, safe ZnO NPs concentrations (5 and 10 μg/ml in smaller and larger sizes of ZnO NPs were obtained, respectively) were treated on the cells for MTT assay and incubated for 72 h at 37 °C (5% CO_2_). The media were removed from the confocal disk after 72 h and were washed with PBS twice and centrifuged. The cells were fixed using ethanol (70%) at 4 °C for 2 days. Then, the incubated cells were again rinsed with PBS and slightly shaken, following which the media were completely removed from the confocal disk. Next, 10 µl RNase was added and incubated for 45 min. For staining, the cells were suspended with 10 µl propidium iodide solution (Sigma, USA). A flow cytometer was sued to evaluate the DNA of cells [36].

### Galactosidase in situ assay for cellular senescence

The activity of senescence-associated-galactosidase (SA-gal), as a common biomarker for aging assay in the cells, was assessed by SA-β-gal staining kit (Thermo Fisher Scientific, Germany) the same as previous studies [37]. Cells were seeded in 24-well plates and treated with two different sizes of ZnO NPs with safe concentrations. Next, they were washed in PBS, fixed in G/F fixative mix (20% glutaraldehyde + 37% formaldyde), and incubated at RT for 3–5 min. Then, the cells were then stained in a fresh staining solution (10 ml citrate Na + 250 μl potassium ferricyanide + 250 μl potassium ferrocyanide + 100 μl MgCl + 250 μl NaCl + 200 μl X-gal) for 2 h in a dark place at 37 °C. SA-β-gal-positive cells exhibiting green color were visualized using an inverted light microscope.

### Real-time PCR

The BMSCs and AMSCs with two different sizes of ZnO NPs were treated in 5 and 10 μg/ml optimum concentrations, respectively. They were harvested after 48 h, and RNA extraction kit (Bio Basic INC) was used to extract total RNA. The first strand of cDNA was synthesized by reverse transcription using SYBR Green qPCR MasterMix 2X kit, SYBR^®^ Premix Ex Taq™ (TaKaRa, Japan). Then, the quality and quantity of the synthesized cDNA was evaluated by a NanoDrop ND-1000 spectrophotometer (Thermo Scientific, Germany). Then, 2 µl of the cDNA was used for target gene amplification. Specific primers were designed by the Primer 3 software and used to amplify the expression of p53, NF-kB, and Nanog genes (Generay Biotech, Korea). The sequencing of primers is presented in Table 1.

**Table 1:** Sequences of primers for real-time PCR analysis of mRNAs

Gene	Primers	Sequence (5′→3′)	Accession number
Nanog	F:	GAGACTGCCTCTCCTCCGCCTT	AB275459.1
	R:	GTGCACACAACTGGGCCTGA	
P53	F:	GTCGGCTCCGACTATACCACTATC	NM_030989.3
	R:	CTCTCTTTGCACTCCCTGGGG	
NF-kB	F:	TTCCCTGAAGTGGAGCTAGGA	NM_199267.2
	R:	CATGTCGAGGAAGACACTGGA	
GAPDH	F:	TCAAGAAGGTGGTGAAGCAG	NM_017008.4
	R:	AGGTGGAAGAATGGGAGTTG	

The expression levels were normalized to the expression level of the GAPDH gene as a housekeeping gene. To perform real-time PCR, the first cycle at 95 °C for 3 min and 40 cycles at 95 °C for 20 s, at 60 °C for 20 s, and at 72 °C for 30 s were used.

### Statistical analysis

All the tests were performed in triplicate, and the results were displayed as mean ± standard deviation (SD). The data were fed into SPSS (IBM Corp. NY, USA) software and analyzed by two-way analysis of variance (ANOVA).

## Results

### Flow cytometry analysis of mesenchymal stem cells

**Fig. 2:**
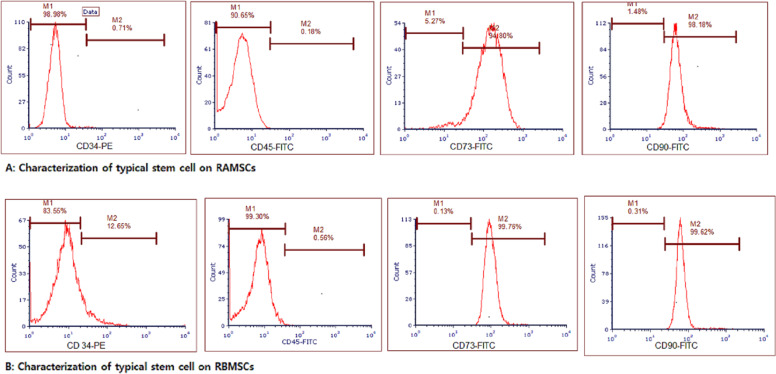
Characterization of the typical stem cell (AMSCs and BMSCs) surface markers CD31, CD34, CD73, and CD90 using flow cytometry. **A** Hematopoietic progenitor cell marker CD34 and pan-leucocyte marker CD45 in BMSCs are ignorable percentages in BMSCs. Surface markers CD73 and CD90 are used as positive markers to identify BMSCs. **B** Hematopoietic progenitor cell marker CD34 and CD45 are low in AMSCs. Surface marker CD90 and CD73 are used as positive markers to identify AMSCs. The *y*-axis represents the cell number and the *x*-axis shows the fluorescence intensity

Rat mesenchymal stem cells were separated from two origins, including adipose tissue and BM. The surface CD markers of MSCs were checked, and a majority of AMSCs or BMSCs (98.18 and 99.62%) were found for CD90 as a positive surface marker in MSCs (Fig. 2A, B). Furthermore, 94.80 and 99.76% of CD73 in AMSCs or BMSCs were definitely stained, respectively (Fig. 2A, B). Moreover, an ignoble percentage of BMSCs or AMSCs showed the expression of CD45 (0.18 or 0.56%) and CD34 (0.71 or 12.65%) in AMSCs or BMSCs, respectively, which are the markers of hematopoietic lineages (Fig. 2A, B). These molecular profiles demonstrate the extraordinary properties of the BMSCs and AMSCs. Furthermore, they approve of the quality removal of hematopoietic cells and isolation of mesenchymal stem cells from adipose tissue and BM during the isolation of stromal cells.

### In vitro cytotoxicity

**Fig. 3:**
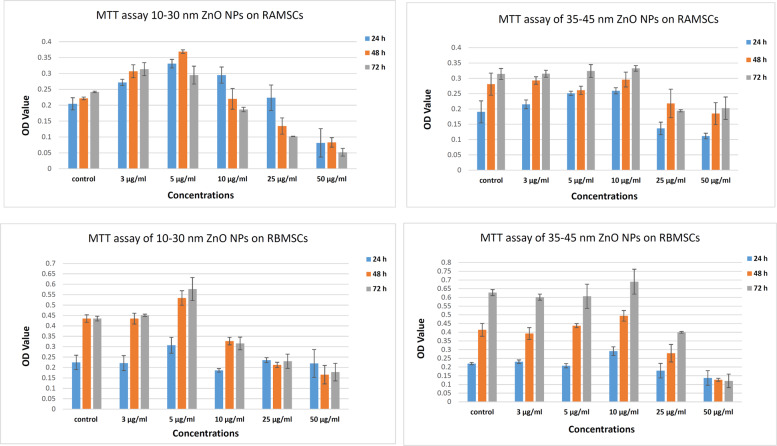
Effects of ZnO NPs on the viability of AMSCs and BMSCs, determined by MTT assay after 1, 2, and 3 days incubation with ~10–30 and 35–45 ZnO NPs in comparison to the control group. Optimum OD of cells treated with 10–30 ZnO NPs was obtained at 5 µg/ml concentration, and optimum OD of cells treated with 35–45 ZnO NPs was obtained at 10 µg/ml concentration. The error bars show mean ± SD

The MTT-based colorimetric cytotoxicity test was applied to investigate the viability of BMSCs or AMSCs following their treatments with 10–30 and 35–45 nm ZnO NPs for 1, 2, and 3 days (Fig. 3). According to the data shown in Fig. 2, the effects of two different ZnO NPs sizes on BMSCs or AMSCs were dependent on dose and time. At dose 5 and 10 µg/ml, 10–30 and 35–45 nm ZnO NPs indicated good viability for the AMSCs and BMSCs, respectively (Fig. 3). Furthermore, the viability of AMSCs and BMSCs with 35–45 nm ZnO NPs was decreased in a dose- and time-dependent manner at the same concentrations (Fig. 3).

### Cell-cycle assay

**Fig. 4:**
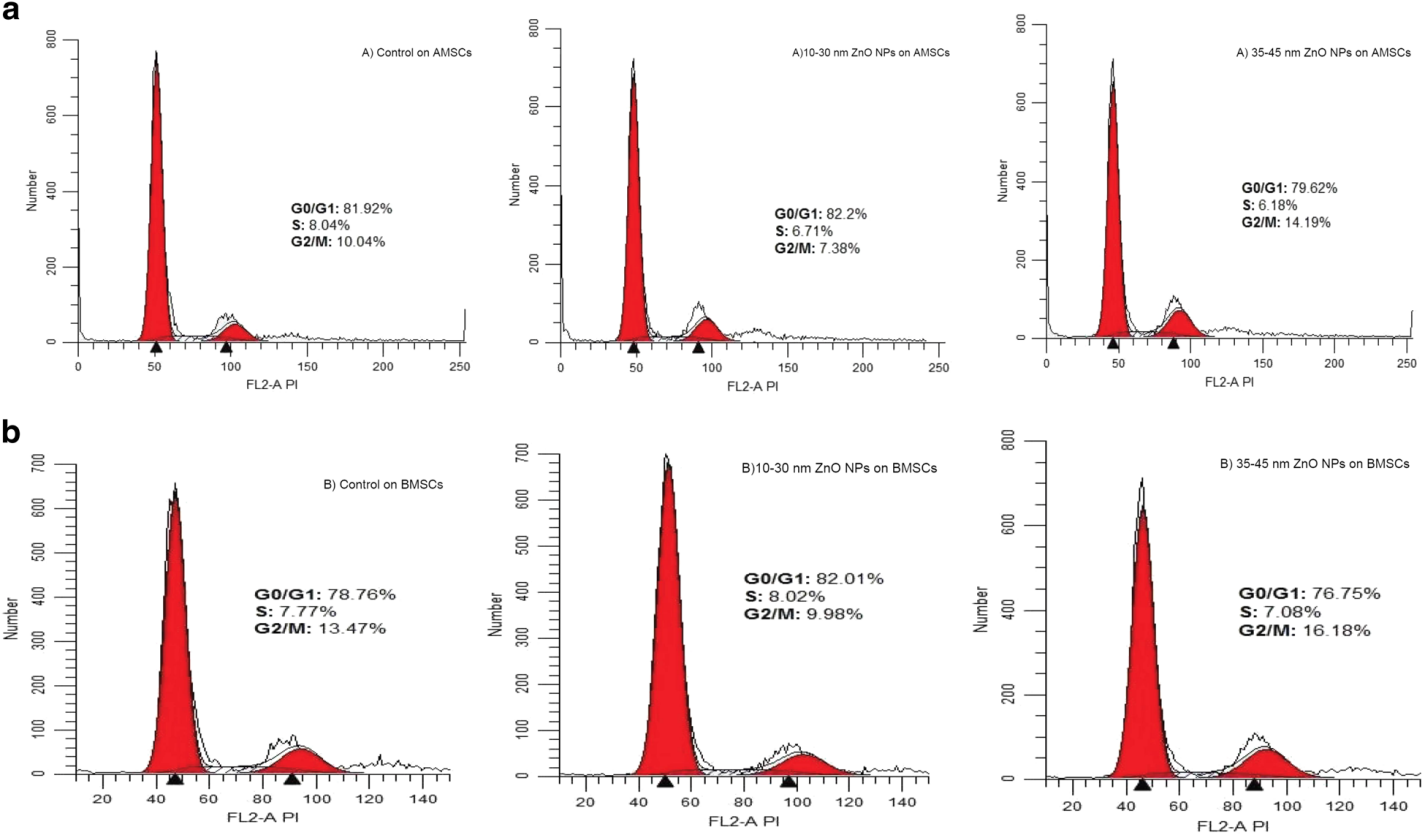
Cell-cycle analysis following flow cytometry of the cells with 5 and 10 µg/ml transfections for 10–30 and 35–45 ZnO NPs, respectively. **a** The AMSCs exposed to 10–30 ZnO NPs represented fewer cells in G2/M as compared to the control group. **b** The BMSCs exposed to 10–30 ZnO NPs represented fewer cells in S and G2/M as compared to the control group

The effects of ZnO NPs on the cell-cycle distributions of BMSCs and AMSCs were studied using propidium iodide staining and measured by flow cytometry. As shown in Fig. 4, the AMSCs and BMSCs treated with 10–30 nm ZnO NPs indicated that the ratio of cells entering G0/G1 phase increased (82.2 and 82.01%) compared to the control group (81.92 and 78.76%, respectively). When signals driving proliferation experience apoptosis or are missing, cells have a tendency to increase in G0/G1 [38].

Moreover, in the AMSCs and BMSCs treated with 10–30 nm ZnO NPs, the G2/M phase decreased (7.38 and 9.98% receptively) compared to the control group (10.04 and 13.47%), indicating that the cells were arrested at S cell-cycle and G2/M phases and were not actively proliferating (Fig. 4). However, cell-cycle analysis of 35–45 nm ZnO NPs indicated an increased accumulation of G2/M phase cells in AMSCs and BMSCs (14.19 and 16.18%, receptively) in comparison to the control group G2/M (10.04 and 13.47%), indicating the retardation of cell-cycle process (Fig. 4). When DNA is harmed, the G2 checkpoint inhibits the mitosis of cells and ensures the proliferation of error-free genome duplicates to each daughter cell.

### Galactosidase in situ assay

**Fig. 5:**
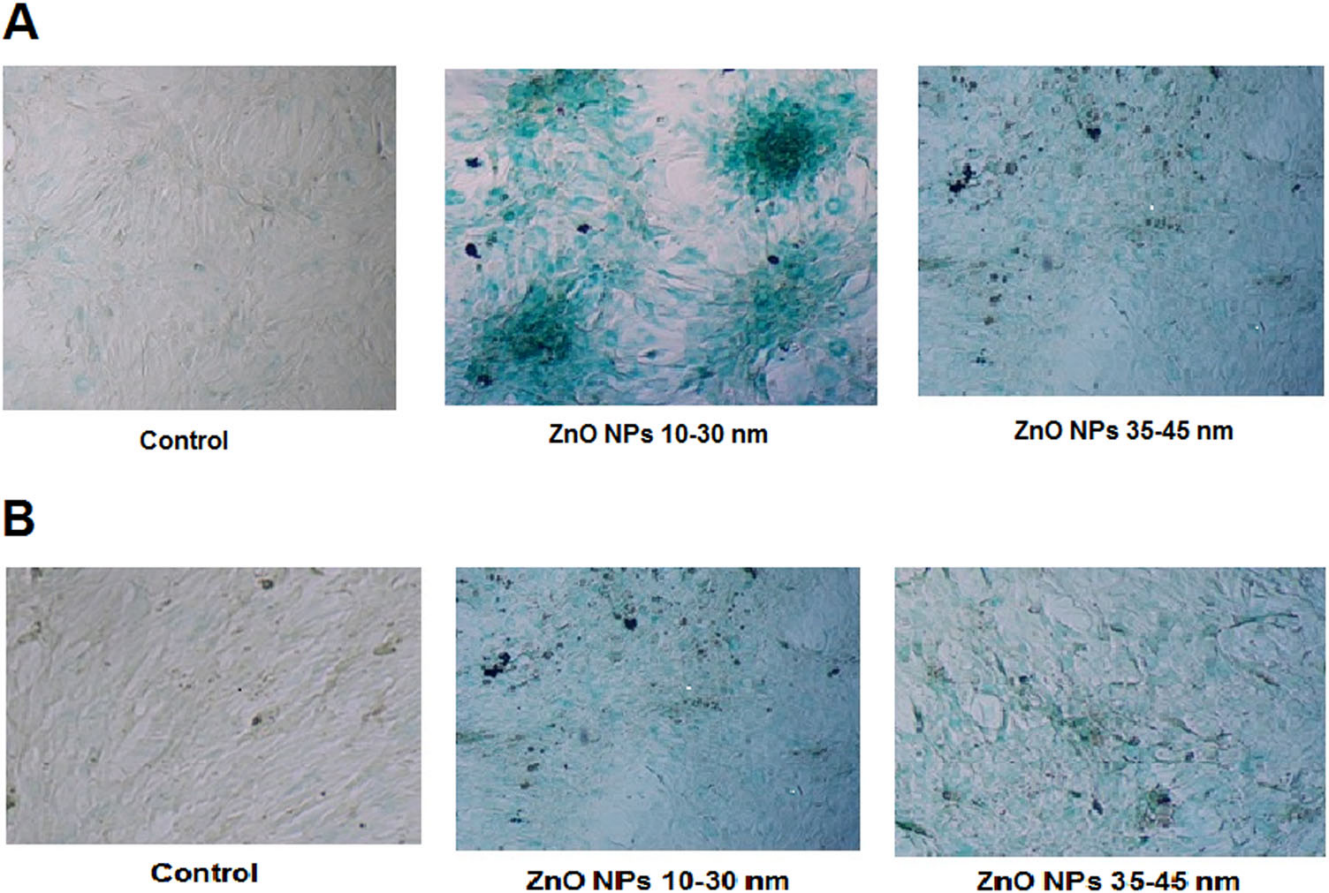
Senescence-associated-β-galactosidase (SA-β-gal) staining in the AMSCs and BMSCs. SA-β-gal-positive cells could be observed more frequently in the treated AMSCs (**A**) and BMSCs (**B**) by ~10–30 and 35–45 ZnO NPs containing 6 and 12 µg/ml concentrations, respectively, in comparison to the control groups

The beta galactosidase enzyme activity was used in this work to check the effect of 10–30 and 35–45 nm ZnO NPs on the senescence of BMSCs and AMSCs. Our results showed that the cell areas with positive green staining were observed more frequently at ZnO NP-treated group in both types of the rat stem cells in comparison with the control group (Fig. 5A, B).

In the normal cells, acid lysosomal β-galactosidases was produced and collected in the lysosome, as observed in the control groups. But in senescent cells, the lysosome increased and produced an upper level of β-galactosidase, named senescence-associated β-galactosidase (SA-β-gal), as detected in the cells treated with ZnO NPs (Fig. 5). Positive cells of SA-β gal were stained blue-green under bright-field microscopy. Both treated cells were positive in comparison to the control groups. The highest blue-green color was obtained in AMSCs with 10–30 nm ZnO NPs (Fig. 5A).

### Real-time PCR

**Fig. 6:**
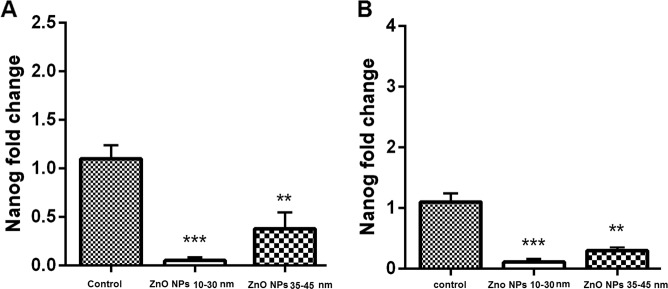
Relative gene expression of Nanog gene in the cells treated with ~10–30 and 35–45 of ZnO NPs in comparison to the control groups. The expression level of the Nanog gene in both AMSCs (**A**) and BMSCs (**B**) was significantly downregulated with 10–30 and ~35–45 nm ZnO NPs (*P* ≤ 0.001 and *P* ≤ 0.01, respectively). The error bars show mean ± SD. ***P ≤ 0.001, **P ≤ 0.01, and *P ≤ 0.05

Relative expression of NF-kB, P53, and Nanog genes was assessed relative to GAPDH as a housekeeping gene on both BMSCs and AMSCs, which were exposed to 5 and 10 µg/ml ZnO NPs in 10–30 and 35–45 nm sizes, respectively, after 48 h. The results of expression of Nanog genes in the cells treated with 10–30 and 35–45 nm ZnO NPs showed significantly (*P* ≤ 0.01) lower regulation in the AMSCs (Fig. 6A) and BMSCs in comparison with the control group (Fig. 6B).

**Fig. 7:**
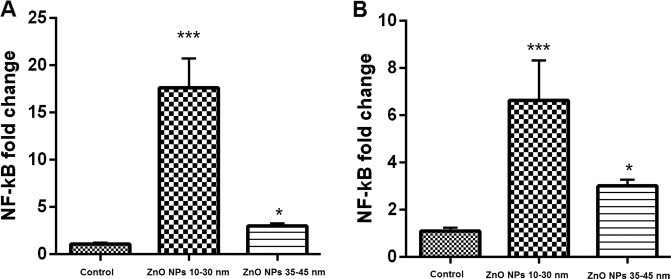
Relative gene expression of NF-kB gene in the cells treated with ~10–30 and 35–45 of ZnO NPs in comparison to the control groups. The expression level of the NF-kB gene in both AMSCs (**A**) and BMSCs (**B**) with ~10–30 and 35–45 ZnO NPs was significantly upregulated (*P* ≤ 0.05 and *P* ≤ 0.001, respectively). The error bars show mean ± SD. ***P ≤ 0.001, **P ≤ 0.01, and *P ≤ 0.05

**Fig. 8:**
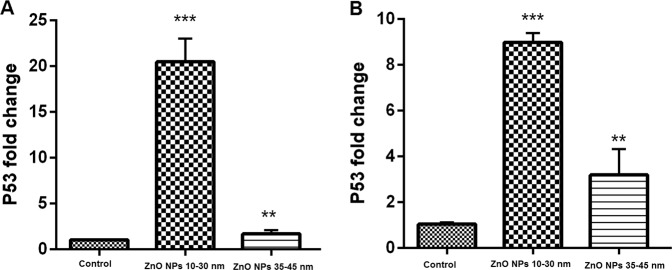
Relative gene expression of the P53 gene in the cells treated with ~10–30 and 35–45 ZnO NPs in comparison to the control groups. The expression level of P53 gene in both AMSCs (**A**) and BMSCs (**B**) with ~10–30 and 35–45 ZnO NPs was significantly upregulated (*P* ≤ 0.001 and *P* ≤ 0.01, respectively). The error bars show mean ± SD. ***P ≤ 0.001, **P ≤ 0.01, and *P ≤ 0.05

The cells treated with 10–30 nm ZnO NPs showed lower expression of Nanog gene compared with the cells treated with 35–45 nm ZnO NPs (Fig. 6). Moreover, the results of expression of NF-kB and P53 genes in AMSCs and BMSCs indicated significant (*P* ≤ 0.01) upregulation compared with the control group (Figs. 7 and 8). As shown in Figs. 6 and 7, the cells treated with 10–30 nm ZnO NPs showed higher overexpression than the cells treated with 35–45 nm ZnO NPs.

## Discussion

This study evaluated the toxic effects of two sizes of ZnO NPs on BMSCs and AMSCs; 10–30 nm as a smaller size and 35–45 nm as a larger size. The results revealed that the smaller size of ZnO NPs had a more toxic effect than its larger size. Surface zone and particle size of NPs are significant material qualities from the toxicological point of view. As the particle size reduces, its surface region elevates and permits a higher extent of its particles or atoms to be shown superficially as opposed to the inner side of the material [39].

Nanomaterials smaller than 50 nm display unique physicochemical properties because of their little size, high surface region, low cost, improved reactivity, and easy entry into the cell dividers [40–42]. According to our MTT assay results, the optimum and safe concentrations of the studied smaller and larger sizes of ZnO NPs were 5 and 10 µg/ml, respectively, in comparison to the control groups. Our cell-cycle analysis showed that safe concentrations of ZnO NPs with 10–30 nm size have toxic effects on AMSCs by decreasing the number of cells in S and G0/G1 phases, indicating the arrest of the cell-cycle process and loss of the signals of drive proliferation. ZnO NPs in 35–45 nm size decreased the cells in G2/M and S phases, resulting in the retardation of cell-cycle process.

**Fig. 9:**
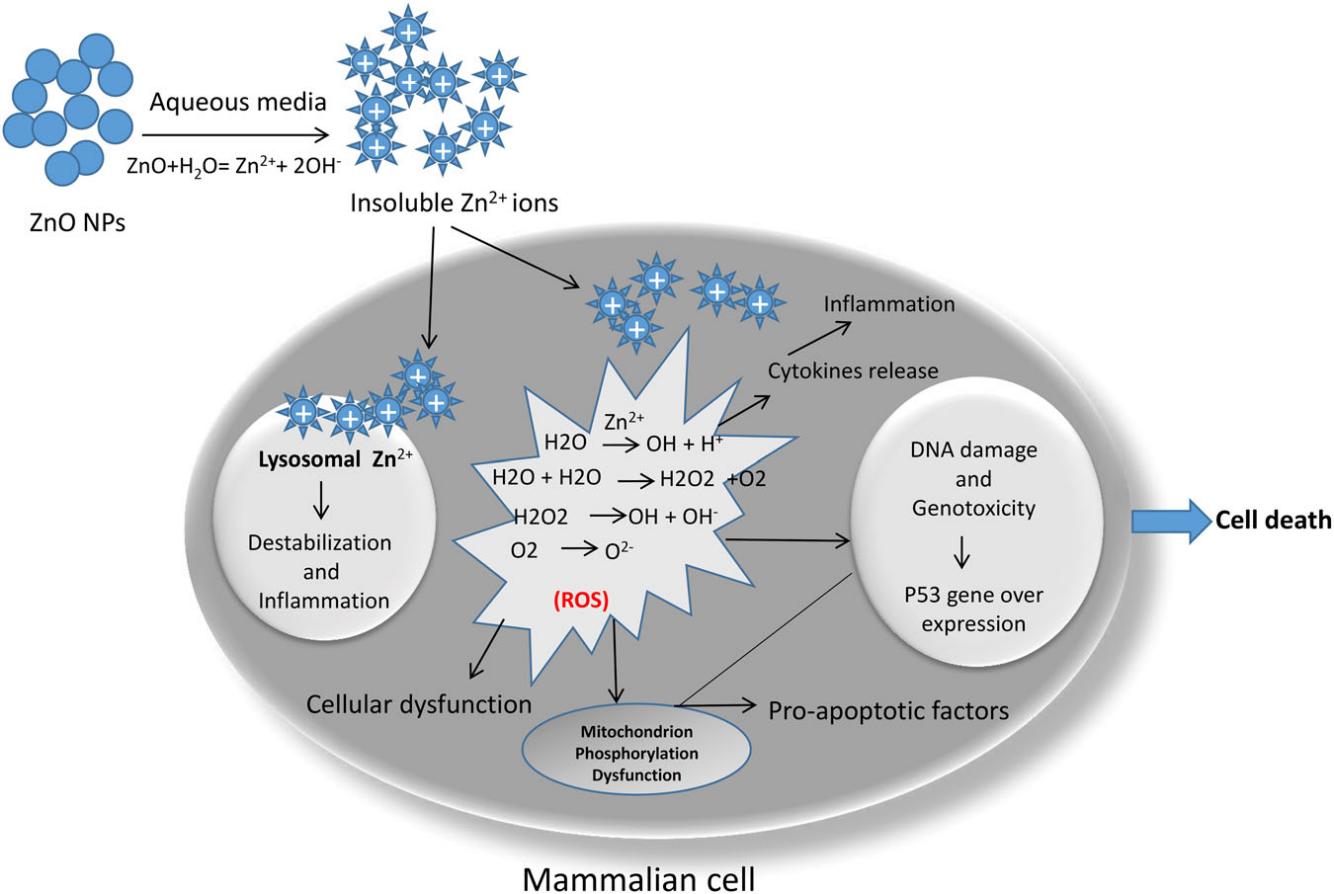
Schematic of ZnO NPs interaction in the mammalian cell. ZnO nanoparticles complex with the water generated Zn^2+^ ions. These ions are insoluble by the cell and generate reactive oxygen species (ROS). ROS resulted in cell inflammation by inducing cytokines and also caused mitochondrion phosphorylation dysfunction and lysosomal destabilization. Finally, ROS caused cellular dysfunction and cell death [10–13]. ZnO NPs influenced genotoxicity and oxidative DNA damage [50, 51]

Our study results are in line with those of other studies that have shown that NPs may lead to death of the cell by means of harming DNA or organelles [43, 44]. Although there are limited toxicological reports of ZnO NPs impact, there are a few reports on ZnO NPs cytotoxic effects in vitro [45–47]. A study showed that oxidative stress was activated in TR146 cells with 10 μg/ml concentration of ZnO NPs [48] and in SH-SY5Y cells with 15 μg/ml concentration [49]. A pro-inflammatory cytokine released in THP-1 cells with 17.69 μg/ml of ZnO NPs [20]. DNA damage was also made at 6.4 μg/ml concentration of ZnO NPs in human colon carcinoma cells [50] and at 12.5 μg/ml concentration in epithelial cells of rat kidney [51]. Contact to nanomaterials is unavoidable because they are becoming a part of our everyday life; accordingly, the toxicity of nanomaterials research is taken into consideration [52]. Figure 9 illustrates the interaction of ZnO NPs in the mammalian cell. The determination of senescent cells showed increased lysosomal β-galactosidase activity level [53]. Our SA-β-gal test results showed that ZnO NPs in both smaller and larger sizes (10–30 and 35–45 nm) stimulated the cells to produce the lysosome. However, a high blue-green color was induced by high lysosomal levels in the cells exposed with 10–30 nm of ZnO NPs in compared with the control group.

P53 works as a lifespan quality of excellence of its strong tumor silencer activity and an aging controller [54]. p53 can decrease and increase oxidative stress possibly due to its double effect on senescence. Our real-time PCR results indicated a significant upregulated expression of p53 and NF-kB genes in the cells exposed to both sizes of ZnO NPs. However, the highest overexpression of these genes was detected in the cells treated with a smaller size (10–30 nm) of NPs. Moreover, the mRNA level of Nanog gene was considered as an anti-aging gene. For Nanog gene, the results of our study showed a significant downregulation of both studied sizes of ZnO NPs in both treated cells. However, the lowest downregulation in the 10–30 nm size indicated that ZnO NPs can be more toxic in the smaller size than in the larger size (35–45 nm).

## Conclusion

ZnO NPs (10–30 and 35–45 nm) prompt the cells to aging process. The smaller size of ZnO NPs has more toxic effects on DNA synthesis than the larger size. The highest β-galactosidase staining was observed in the smaller size than the larger size of ZnO NPs. In addition, a significant overexpression of aging-related genes (NF-kB and p53) was acquired in ZnO NPs cells treated with both sizes. Furthermore, a significant downregulation of anti-aging-related gene (Nanog) was acquired in the cells treated with ZnO NPs.
